# HIV knowledge, attitudes and practices amongst patients with severe mental illnesses and chronic medical illnesses in Durban, South Africa

**DOI:** 10.4102/sajpsychiatry.v27i0.1586

**Published:** 2021-06-28

**Authors:** Thembeka Matshoba, Sibongile Mashaphu, Andrew Tomita, Saeeda Paruk

**Affiliations:** 1 Department of Psychiatry, School of Clinical Medicine, University of KwaZulu-Natal, Durban, South Africa; 2 Centre for Rural Health, School of Nursing and Public Health, University of KwaZulu-Natal, Durban, South Africa; 3 KwaZulu-Natal Research Innovation and Sequencing Platform (KRISP), College of Health Sciences, University of KwaZulu-Natal, Durban, South Africa

**Keywords:** HIV, human immunodeficiency virus, AIDS, acquired immune deficiency, knowledge, attitude, practices, South Africa

## Abstract

**Background:**

Studies exploring HIV knowledge, attitudes and practices (KAP) of individuals with severe mental illness (SMI) have suggested their poorer knowledge about HIV. In KwaZulu-Natal (KZN) province, South Africa (SA), the epicentre of the country’s HIV epidemic, improving KAP is essential for reduce its incidence amongst individuals with SMI. Comparing the KAP related to HIV between those with SMI and chronic medical illnesses (CMI) such as hypertension and diabetes may expose gaps in KAP related to HIV in the mentally ill who are more vulnerable to HIV.

**Aim:**

This study aimed to compare the KAP related to HIV between people living with SMI and CMI.

**Setting:**

Outpatient clinics in Durban, SA.

**Methods:**

A cross-sectional structured questionnaire survey was conducted amongst 214 adult outpatients with SMI and CMI attending two general public sector hospitals in Durban, KZN. The KAP questionnaire consisted of three sections: general information, prevention and transmission of HIV.

**Results:**

Interviews were conducted with 124 patients with SMI and 90 with CMI. Most were female (69.5%), single (57.5%) and unemployed (59.4%). The diagnosis of SMI was associated with poorer general information of HIV (*p* = 0.02), but not with its prevention and transmission compared with those with CMI. Educational level was associated with poorer performance in all three domains: general information of HIV (*p* = 0.01), prevention (*p* = 0.01) and transmission (*p* = 0.02) amongst all the participants.

**Conclusion:**

Gaps in the KAP of HIV amongst individuals with SMI compared with those with CMI suggested a need to provide focused health promotion regarding sexual health and HIV to the mentally ill at psychiatric facilities.

## Introduction

Global epidemiological studies have reported that people with severe mental illness (SMI) may be disproportionately affected by HIV.^1,2,3^ This is particularly of concern in South Africa (SA), which has the largest number of people infected with HIV worldwide, with KwaZulu-Natal (KZN) province having the highest incidence in the country.^4,5^ Studies exploring HIV knowledge, attitudes and practices (KAP) of individuals with SMI have often pointed to the lack of knowledge about HIV transmission and risk prevention in this group of vulnerable individuals.^2,3,6^

The mentally ill population is faced with numerous challenges, such as stigma, exploitation and unemployment.^7^ Severe mental illness is generally associated with low self-worth and impaired judgement during active illness, relapses or substance abuse, which often prevent the mentally ill from negotiating or practicing safe sex, making this population more likely to engage in high-risk relationships, including with HIV-infected individuals, to avoid social isolation.^8,9^

A South African study in Gauteng in 2011 reported that mentally ill patients had poor knowledge about many aspects of HIV and harboured several myths associated with its transmission.^9^ The general perceptions of some black South Africans attributed HIV prevalence to witchcraft, genocide and other conspiracy theories.^10^ The mentally ill shared similar beliefs but were unsure about HIV acquisition and believed that it can be contracted by both direct or indirect forms of physical contact and even mosquito bites.^9^ These gaps in knowledge were particularly prevalent amongst older and less educated patients.^9^

Other factors, such as being single, male gender, low education levels with less than 5 years of schooling and a diagnosis of severe and chronic mental illness, such as schizophrenia, were associated with limited knowledge on HIV transmission.^11^ Inadequate knowledge about HIV remains a challenge in the post highly active antiretroviral (HAART) drugs era, with people acquiring information from a variety of sources, such as the media, family and friends and a small proportion from the healthcare systems.^9^ Hence, the source of information may influence the reliability of KAP related to HIV.

An important step in identifying the service development needs of people with SMI is to establish their KAP in relation to sexual health promotion and HIV prevention routine care.^12,13^ The assumption is that correct knowledge results in relevant attitudes and appropriate behaviour. By redefining HIV as an educational and behavioural problem that can be controlled by modifying high-risk behaviour, people can be empowered to control the disease.^14,15^ Previous studies on HIV and high-risk behaviour in patients with SMI have consistently recommended the inclusion of HIV health education in their treatment and care.^3,16,17^

This study examined the knowledge, attitudes and practices of people living with SMI and compared them with those of people with chronic medical illnesses (CMI) such as hypertension, diabetes and asthma in the post antiretroviral era as both groups are regularly attending healthcare services but may receive different health education. We also assessed the perception of the patients with SMI attending psychiatric clinics about the availability of HIV health education at these facilities.

## Methods

### Study design

A cross-sectional questionnaire survey of randomly selected patients with SMI and CMI attending two general public sector hospitals in Durban, KZN province, SA, was conducted.

Data were collected from October 2017 to April 2018 from the specialist psychiatric and general medical outpatient clinics at both hospitals. A comparative approach was used, with patients with SMI categorised as the study group and those with other CMI as the control group. Adult participants who had been attending for at least 6 months were aged from 18 to 65 years and gave consent to participate were recruited at the clinics. Pre-screening interviews to establish participants’ eligibility were conducted by the principal investigator (PI) and written informed consent was obtained. Participants who required urgent acute medical or psychiatric care were referred appropriately and excluded from the study. The interviews were conducted in the participant’s language of preference (English or IsiZulu) by the bilingual PI.

### Measures

The participants were interviewed using a structured sociodemographic questionnaire and a self-designed 50 item standardised questionnaire to establish their KAP related to HIV, with additional questions related to a review of the current literature and available tools. The 50-item KAP questionnaire was subdivided into five sections: (1) sociodemographic and clinical variables, that is, age, gender, race, marital status, employment, educational level, facility and diagnosis (mental or medical illness), (2) their KAP about general HIV information, (3) prevention, (4) transmission measures and (5) additional general comments.

The KAP indicators were based on the recommendations of the Basic Indicators of the Joint United Nations Programme on HIV and AIDS (UNAIDS), and previous studies which were conducted at health care providers.^18^ The KAP questionnaire was based on existing instruments developed for use in nursing and complemented by items obtained from the literature adapted to the local context.^19^

Quantitative data were collected to ensure standardisation of the interviews that consisted of ‘Yes’, ‘No’ or ‘I don’t know’ for each item and recorded on coded data sheets, with correct responses = 0 and incorrect and unknown responses = 1. For patients attending the psychiatric clinics, section 5 (additional general comments) included eight additional questions on their perception of HIV education and health sharing information at their psychiatric health facility using the same response options. Finally, there were also four quantitative questions related to their perception towards HIV, fidelity in sexual relationships and condom use, which all respondents could agree or disagree with using a yes or no response.

### Data analysis

The analysis consisted of five components: the first component entailed descriptive statistics of the participants’ sociodemographic information. Secondly, the KAP items related to their general HIV information, prevention and transmission measures were individually analysed for the two groups (SMI and CMI). In the third component, the response to the questions about HIV and KAP was compared using Chi-square statistics. In the fourth component the data on perception of HIV health promotion and care services were analysed amongst the SMI patients, using descriptive statistics. In the fifth component, three fitted bivariate/multiple regression models were used to assess the difference in their knowledge about and prevention and transmission of HIV between individuals with SMI and CMI. Given the skewness of the knowledge score about HIV and its prevention and transmission, cubic data transformation was applied to normalise the distribution. Owing to the large number derived from the cubic data, the transformed value was divided by 10 000 for readability. The data were analysed by using STATA 15.

### Ethical considerations

The University of KwaZulu-Natal Biomedical Research Ethics Committee (BREC reference number: BE187/17) approved the research protocol. Permission to conduct the research was also obtained from the medical managers of the two hospitals and the KZN Provincial Department of Health. Written informed consent was obtained from all the study participants. Participants who requested HIV counselling or testing during the survey were referred appropriately.

## Results

### Sociodemographic profile

During the 7 months, 214 participants were interviewed, of whom 124 (58%) had an SMI and 90 (42%) had a CMI with no psychiatric history. The majority were females (*n* = 153, 71%), with the largest age category being 46 years and older (*n* = 89, 39.7%) (Table 1). The majority were black people (*n* = 165, 75%), single (*n* = 127, 58%), unemployed (*n* = 129, 59%) and collecting a disability grant (*n* = 32, 15%). The most common CMI diagnosis was hypertension (*n* = 33, 29.7%), diabetes mellitus type II (*n* = 34, 30.6%) and asthma (*n* = 10, 9%). The most prevalent SMIs were major depressive disorder (*n* = 29, 35.9%), schizophrenia (*n* = 24, 29.8%) and bipolar mood disorder (*n* = 18, 22.3%).

**TABLE 1:** Sociodemographic and clinical characteristics of participants.

Demographics and site	Overall (*n* = 214)		Chronic medical illness *n* = 90 (42%)		Severe mental illness *n* = 124 (57.9%)	
	*n*	%	*n*	%	*n*	%
**Gender**						
Female	153	71.5	71	78.9	76	61.3
Male	67	28.5	19	21.1	48	38.7
**Employment status**						
Employed	56	-	28	31.5	28	23.0
Unemployed	129	-	55	61.8	68	55.7
Disability	32	-	6	6.7	26	21.3
**Age category**						
18–24	24	-	9	10.0	15	12.1
25–34	57	-	18	20.0	38	30.6
35–45	54	-	21	23.3	32	25.8
46 +	85	-	42	46.7	39	31.5
**Ethnicity**						
African	165	-	70	77.8	90	72.6
Mixed race	13	-	5	5.6	8	6.5
Indian	28	-	14	15.6	13	10.5
White people	14	-	1	1.1	13	10.5
**Education**						
Primary level (up to Grade 7)	33	-	14	15.9	17	14.0
Secondary level (Grade 8 to 12)	131	-	50	56.8	78	64.5
Tertiary level (degree, diploma and certificate)	50	-	24	27.3	26	21.5
**Marital status**						
Single	127	-	49	54.4	75	60.5
Married or co-habitation	65	-	30	33.3	32	25.8
Divorced, widowed and separated	28	-	11	12.2	17	13.7
**Facility**						
Medical 1	55	-	0	0.0	53	42.7
Medical 2	55	-	44	48.9	8	6.5
Psychiatric 1	55	-	1	1.1	54	43.5
Psychiatric 2	55	-	45	50.0	9	7.3

### Knowledge, attitudes and practices on HIV

Table 2 summarises the participants’ response to questions on their KAP about HIV. The majority answered correctly on general information about HIV and its prevention and transmission, although 26 (20.0%) participants with SMI did not know that HIV was caused by a virus compared with 15 (13.5%) with CMI, which was not statistically significant. Regarding determining an individual’s HIV status based on their physical appearance, those with SMI had more incorrect responses (*n* = 29, 23%) than those without mental illness (*n* = 8, 9%) (*p* = 0.01). The most incorrectly answered questions were related to ART (‘with the new HIV medication, HIV is no longer a danger’) and viral load (‘if a person’s viral load is undetectable, they cannot transmit HIV’) by all the respondents, with no statistically different responses to these questions between the two groups.

**TABLE 2:** Participants responses to general information, prevention and transmission of HIV.

HIV knowledge, attitudes and practices	All groups (*n* = 214)		Chronic medical illness (*n* = 90)		With severe mental illness (*n* = 124)		χ^2^	*p*
	Correct response		Correct response		Correct response			
	*n*	%	*n*	%	*n*	%		
**General information on HIV**								
HIV is caused by a virus	178	83.6	75	86.2	98	81.7	0.76	0.38
HIV is caused by a curse	171	79.5	74	83.1	94	78.3	0.75	0.39
HIV is caused by punishment from God	183	85.5	81	90.0	99	83.9	1.63	0.20
HIV is caused by being bewitched	196	91.6	82	93.2	108	90.0	0.65	0.42
Traditional healers can cure HIV	188	85.8	76	85.4	108	87.1	0.13	0.72
Sex with a virgin can cure HIV	201	93.1	82	92.1	113	93.4	0.12	0.73
Eating healthy food can cure HIV	164	75.9	67	76.1	93	76.2	0.00	0.99
Only people who look sick can spread HIV	157	74.1	70	81.4	84	70.0	3.45	0.06
Most people with HIV show signs of being sick right away	138	63.3	60	67.4	75	61.0	0.93	0.34
Antiretroviral medication can cure HIV	138	63.6	52	59.1	82	66.7	1.27	0.26
I can tell if someone has HIV by looking at them	182	83.5	82	91.1	95	77.9	6.59	0.01
People living with HIV can become infected with a different strain	118	54.6	53	61.6	60	48.4	3.58	0.06
With the new HIV medication, HIV is no longer a danger	80	37.4	32	36.8	47	38.8	0.09	0.76
Having sex with someone who has HIV is the only way of becoming infected	152	69.7	66	73.3	83	68.0	0.70	0.40
If a person’s viral load is undetectable, they cannot transmit HIV	97	45.1	43	50.0	50	40.7	1.79	0.18
**Prevention of HIV**								
Female condoms are effective in preventing HIV	130	60.2	54	61.4	72	59.0	0.12	0.73
Not having sex is a good way to prevent HIV	162	73.6	64	71.1	94	75.8	0.6	0.44
Birth control pills can prevent HIV	181	83.8	74	85.1	101	82.1	0.32	0.57
Using condoms can prevent HIV	178	89.9	73	88.0	99	90.8	0.42	0.52
Traditional medicines can prevent HIV	172	78.9	69	78.4	98	79.0	0.01	0.91
A healthy diet can prevent HIV	144	66.4	64	73.6	77	62.1	3.03	0.08
Regular induced vomiting can prevent HIV	195	90.7	83	94.3	107	88.4	2.14	0.14
Only people with HIV use condoms	195	89.0	83	93.3	107	86.3	2.61	0.11
You should use a condom when you have sex with your regular partner	156	71.9	63	70.8	87	71.3	0.01	0.93
You should use a condom when you have more than one sexual partner	212	96.4	88	97.8	118	95.2	0.99	0.32
In the absence of a condom, I will not have sex	151	69.9	62	70.5	84	68.9	0.06	0.80
Washing yourself after sex can prevent HIV	172	78.5	72	80.9	95	76.6	0.56	0.45
You can acquire HIV if you have sex just once without using a condom	191	86.8	79	87.8	106	85.5	0.23	0.63
Antiretrovirals can prevent babies from being born with HIV	131	59.8	60	66.7	67	54.5	3.21	0.07
**Transmission of HIV**								
One can acquire HIV from a mosquito bite	153	69.9	62	69.7	86	69.4	0.00	0.96
Only people who have sex with gay people acquire HIV	110	50.2	48	53.9	57	46.0	1.32	0.25
One can acquire HIV from dirty toilet seats	144	65.8	62	69.7	79	63.7	0.82	0.37
Having many sexual partners increases the risk of acquiring HIV	209	95.0	86	95.6	118	95.2	0.02	0.89
Other people can infect you deliberately	185	84.5	81	90.0	100	81.3	3.08	0.08
One can acquire HIV by sharing a hospital ward with infected people	170	77.6	76	85.4	93	75.0	3.41	0.07
Using unclean razor blades for traditional scarification can transmit HIV	166	76.2	64	71.1	99	81.1	2.94	0.09
HIV-positive breastfeeding mothers can transmit HIV to their babies	123	55.9	45	50.0	73	58.9	1.66	0.20

Source: Adapted from Kaladharan S, Daken K, Mullens AB, Durham J. Tools to measure HIV knowledge, attitudes practices (KAPs) in healthcare providers: A systematic review. AIDS Care. 2020:1–7; Delobelle P, Rawlinson JL, Ntuli S, Malatsi I, Decock R, Depoorter AM. HIV/AIDS knowledge, attitudes, practices and perceptions of rural nurses in South Africa. J Advanced Nurs. 2009;65(5):1061–1073.

### Patient perception of HIV information and care at psychiatric clinics

Table 3 presents the attitudes and perceptions to HIV information and care received by participants with SMI. A total of 58 (47%) of the 124 participants attending the psychiatric outpatient clinic perceived health information sharing and education about HIV to be inadequate and the same number indicated that they did not know, despite HIV being recognised as essential in this setting. In addition, 93 (79%) of those with SMI believed that HIV can cause psychological/psychiatric problems, whilst 74% felt that people with mental illness were at risk of acquiring HIV infection.

**TABLE 3:** Perception of patients with severe mental illness on HIV services at psychiatric clinics.

SMI perceptions	Yes		No		Do not know	
	*n*	%	*n*	%	*n*	%
One can acquire information about HIV from the psychiatric clinic	66	55.9	36	30.5	16	13.6
HIV counselling is available in psychiatric clinics	58	49.2	38	32.2	22	18.6
People with mental illness must know about HIV	108	91.5	5	4.2	5	4.2
People with mental illness can be tested for HIV if they wish to do so	107	92.2	4	3.4	5	4.3
People with mental illness are at risk of acquiring HIV infection	88	74.6	23	19.5	7	5.9
HIV can cause psychological/psychiatric problems	93	78.8	7	5.9	18	15.3
People with mental illness can take antiretroviral medication	104	87.4	6	5	9	7.6

Source: Adapted from Kaladharan S, Daken K, Mullens AB, Durham J. Tools to measure HIV knowledge, attitudes practices (KAPs) in healthcare providers: A systematic review. AIDS Care. 2020:1–7; Delobelle P, Rawlinson JL, Ntuli S, Malatsi I, Decock R, Depoorter AM. HIV/AIDS knowledge, attitudes, practices and perceptions of rural nurses in South Africa. J Advanced Nurs. 2009;65(5):1061–1073.

Note: Those with chronic medical illnesses were not surveyed for these questions.

Regarding the four additional questions with no correct or incorrect answer related to perception towards HIV, fidelity in sexual relationships and condom use, 113 (52%) of all the respondents concurred with the statement that people with HIV are subjected to harsh treatment, whilst 146 (67%) believed that partner fidelity protects one from HIV. Access to condoms was viewed as easy by the majority of respondents (*n* = 204, 95%), with 20% indicating that they were not comfortable using them.

### Association of knowledge, attitudes and practices with sociodemographic and clinical variables

The results of the three fitted bivariate/multiple regression models indicate that diagnosis of SMI and secondary level of education were associated with poor knowledge about HIV, whilst female gender was associated with better knowledge on HIV prevention strategies. Table 4 shows the regression results for KAP, which were subdivided into general HIV information, prevention and transmission. Table 5 shows an additional analysis for variables that were significant in Table 4.

**TABLE 4:** Sociodemographic and clinical comparison of participant knowledge, attitudes and practices to HIV responses.

Variable	KAP score – Information on HIV				KAP score – HIV prevention				KAP score – HIV transmission			
	Β	Adjusted β	SE	*p*	β	Adjusted β	SE	*p*	β	Adjusted β	SE	*p*
**Diagnosis: No mental illness**												
Severe mental illness	−6.03	−16.07	6.55	0.02	−4.93	−12.36	6.84	0.07	−0.89	−2.90	7.61	0.70
Chronic medical illness	-	-	-	-	-	-	-	-	-	-	-	-
**Gender: Female**												
Male	−2.39	−0.60	3.89	0.88	−8.51*	−7.28	4.06	0.08	−4.71	−6.15	4.52	0.18
**Employment: Employed**												
Unemployed	−6.27	−6.33	4.14	0.13	−1.25	−1.53	4.33	0.72	−1.21	−1.11	4.81	0.82
Disability pension	−15.77**	−14.83	5.95	0.01	−15.01*	−12.08	6.21	0.05	−5.15	−5.02	6.91	0.47
**Age category: 18–24 years**												
25–34	−0.75	−0.91	6.07	0.88	2.36	3.18	6.33	0.62	−13.76*	−12.06	7.04	0.09
35–45	4.75	3.81	6.22	0.54	13.16*	13.65	6.50	0.04	−3.85	−5.02	7.23	0.49
46+	−1.39	−0.64	6.31	0.92	1.17	3.46	6.59	0.60	−10.19	−13.42	7.33	0.07
**Ethnicity: White people**												
African	−8.83	−9.38	7.71	0.23	2.61	4.10	8.05	0.61	−15.33	−10.97	8.95	0.22
Mixed race	−7.55	−4.85	10.28	0.64	1.07	10.46	10.73	0.33	4.43	6.68	11.94	0.58
Indian	−9.38	−9.76	8.80	0.27	0.74	6.20	9.19	0.50	−8.71	−3.62	10.22	0.72
**Education: Up to Grade 7**												
Secondary level (Grade 8 to 12)	13.56**	9.30	5.07	0.07	8.76	8.45	5.29	0.11	13.33*	11.22	5.89	0.06
Tertiary level	21.75***	15.85	5.89	0.01	17.17**	14.19	6.15	0.02	20.95**	16.97	6.84	0.01
**Marital status: Single**												
Married or co-habitation	0.69	0.70	4.57	0.88	1.59	0.75	4.77	0.88	6.15	5.51	5.30	0.30
Divorced, widowed and separated	3.85	4.03	6.01	0.50	−10.92*	−11.52	6.28	0.07	0.51	−6.26	6.98	0.37
**Facility: Medical 1**												
Medical 2	2.19	−16.04	7.87	0.04	−1.45	−17.74	8.21	0.03	−1.32	−5.88	9.14	0.52
Psychiatric 1	−4.19	−5.81	5.07	0.25	−9.13	−10.82	5.30	0.04	−11.24*	−8.39	5.89	0.16
Psychiatric 2	−1.61	−15.80	7.73	0.04	−2.87	−16.11	8.06	< 0.05	−10.47	−8.04	8.97	0.37

KAP, knowledge, attitudes and practices.

* , *p* ≥ 0.05;

** , *p* < 0.05;

*** , *p* < 0.01 for bivariate analyses.

**TABLE 5:** Sociodemographic and clinical comparison of participant knowledge, attitudes and practices to HIV/AIDS responses stratified by chronic medical illness and severe mental illness.

Variable	Chronic medical illness									Severe mental illness								
	KAP score Information on HIV			KAP score HIV prevention (Chronic medical illness)			KAP score HIV transmission (Chronic medical illness)			KAP score Information on HIV (Severe mental illness)			KAP score HIV prevention (Severe mental illness)			KAP score HIV transmission (Severe mental illness)		
	Adjusted β	SE	*p*	Adjusted β	SE	*p*	Adjusted β	SE	*p*	Adjusted β	SE	*p*	Adjusted β	SE	*p*	Adjusted β	SE	*p*
**Gender: Female**																		
Male	4.46	6.13	0.47	−6.90	7.11	0.33	−9.77	7.22	0.18	−6.13	4.79	0.20	−8.62	4.69	0.07	−5.44	5.79	0.35
**Employment: Employed**																		
Unemployed	−15.72	5.42	< 0.01	−8.18	6.29	0.20	−9.26	6.38	0.15	5.23	5.80	0.37	8.41	5.69	0.14	9.30	7.02	0.19
Disability pension	−5.48	11.26	0.63	4.76	13.05	0.72	−2.73	13.26	0.84	−8.27	7.16	0.25	−9.22	7.03	0.19	2.64	8.67	0.76
**Age category: 18–24 years**																		
25–34	3.37	9.53	0.72	12.68	11.06	0.26	2.48	11.23	0.83	−7.03	7.67	0.36	−2.58	7.53	0.73	−19.56	9.28	0.04
35–45	11.06	9.38	0.24	28.86	10.88	0.01	8.37	11.05	0.45	−3.17	7.87	0.69	3.47	7.72	0.65	−8.36	9.52	0.38
46+	2.62	8.77	0.77	16.37	10.17	0.11	3.84	10.33	0.71	−5.31	7.75	0.49	−9.45	7.60	0.22	−14.41	9.37	0.13
**Education: Up to Grade 7**																		
Secondary level (Grade 8 to 12)	−0.20	7.34	0.98	10.81	8.51	0.21	15.68	8.64	0.07	13.42	7.02	0.06	4.34	6.89	0.53	8.82	8.49	0.30
Tertiary level	10.95	8.17	0.18	14.30	9.47	0.13	20.64	9.62	0.04	14.32	8.37	0.09	7.53	8.21	0.36	15.15	10.12	0.14

The facility variable was not included in the model because of sample size limitation.

KAP, knowledge, attitudes and practices.

## Discussion

This study investigated the KAP about HIV amongst a sample of people with SMI and compared them with people living with CMI. This was carried out as people living with SMI have increased prevalence of HIV compared with the general population.^20,21^ The diagnosis of SMI was associated with poorer general information on HIV (*p* = 0.02) but not on HIV transmission or prevention (Table 2).

People with SMI were more likely to believe in the ability to determine HIV status by physical appearance alone (Table 2) and perceived HIV health information sharing and education at psychiatric facilities to be inadequate (Table 3). Educational level was associated with poorer performance in all three domains, namely, general information of HIV (*p* = 0.01), transmission (*p* = 0.02) and prevention (*p* = 0.01) for both groups as a total (Table 4) and for knowledge on HIV transmission when the two groups were compared (Table 5).

### Knowledge, attitudes and practices on HIV

The results of this study demonstrate a possible association between SMI diagnosis and poor general knowledge about HIV when compared with participants with CMI (Table 4). A South African study on HIV amongst mentally ill patients in Soweto reported similar results, but lacked the comparative group.^9^ Peltzer and colleagues conducted a study on the general population in SA and concluded that media and mass public communication in the area of HIV transmission were effective.^22^

At least 70% of all the participants reported that they would not engage in any sexual activities in the absence of a condom, with no statistical difference between the groups. Previous studies have indicated that people with mental illness and substance abuse may be more vulnerable to HIV because of inappropriate decision-making.^20,21^ This was affected by illness-related behaviours such as hyper-sexuality; external factors, for example, comorbid substance use, with intoxication resulting in poor decision-making about safe sex; lack of assertion about insisting on condom use; vulnerability to coercion for sex and selling or swapping sex for cash.^23^ These findings suggest that appropriate health information about HIV should form an integral part of the treatment and care of people with SMI.

### Patient perception of HIV information and care at psychiatric clinics

The study’s participants demonstrated an appropriate appreciation that people with mental illness should receive counselling, health information and voluntary testing for HIV. However, a few misconceptions were identified, with 80% of all participants believing that traditional medicines could prevent HIV (and no statistical difference between the two groups), which is consistent with another local study.^24,25^ In some African communities, it is not uncommon for people to associate traditional healers with the ability to treat or cure HIV.^15,25^ There is also strong evidence for male circumcision as an effective prevention measure for HIV, which is incorporated into an ongoing cultural practice of young male circumcision that is facilitated by traditional African leaders.^26^ Education and support for traditional community leaders or counsellors is essential to ensure the implementation of effective HIV prevention strategies.^27^

Another common misconception identified amongst all the participants was that washing yourself after sex could prevent HIV. A study performed in sub-Saharan Africa reported that genital hygiene cleaning has long been regarded as being protective against sexually transmitted infections.^10,28^ The authors noticed that whilst genital hygiene after sex may be common in this region, HIV prevention programmes should address local misconceptions and other inappropriate practices in a culturally sensitive manner.^28,29^

Our study findings indicated that participants with SMI more likely believed that they could tell if someone has HIV from their physical appearance than the CMI group. This finding is consistent with previous studies on the general population, in which participants displayed misconceptions and believed that they could identify HIV status by physical appearance.^30,31^ These findings are especially relevant in the SMI participants with poor KAP on HIV, as such misconceptions may result in risky behaviours.^9^ A previous local study on HIV amongst the general population established an association between high-risk sexual behaviours and beliefs, such as that HIV was caused by witchcraft or poor dietary intake.^10^

### Patient perception of HIV health education at psychiatric clinics

This study finding of the perceived lack of HIV education or information sharing in psychiatric settings (Table 3) is concerning. It is important to reinforce knowledge on the role of highly active antiretroviral treatment in lowering disease progression, preventing new infections and improving the lives of those infected and affected by HIV.^32^ Therefore, HIV screening and dissemination of relevant information should form part of the assessment, routine care and follow-up of the mentally ill. It is also important to develop prevention programmes that not only focus on providing information but also correct and dispel incorrect information and misconceptions. There is evidence from controlled trials that high-risk behaviour can be reduced in people with SMI with targeted behavioural skills,^13,33^ with culturally and linguistically relevant interventions needing to be developed and implemented.

Finally, the finding that poorer KAP on HIV and AIDS was associated with educational level for general information, prevention and transmission of HIV (Table 4) is consistent with the literature both locally and internationally.^9,34^ Hence, this suggests that health promotion and disease reduction strategies require broader societal challenges to be addressed, such as improving education and empowering patients to decrease unemployment and reduce poverty.

## Limitations

This study had a number of limitations, including that it was cross-sectional and conducted at two general hospitals, which means that it therefore cannot be generalised to other community populations. The self-reporting nature of the questionnaire could have led to bias in answering the questions. The lack of objective HIV testing of participants might have also created bias. The lack of a standardised validated tool to measure KAP towards HIV also limits comparison with the literature. Finally, the small sample size might have also limited the capacity to assess for associations adequately.

Further research is required to establish where people obtain their HIV information from and then we need to target the community and clinic sources with appropriate information.

## Conclusion

Whilst participants diagnosed with SMI and chronic medical illness were generally well-informed about HIV, misconceptions in the former were identified. The findings suggest the need to enhance HIV prevention and transmission knowledge at mental healthcare services to better equip this at-risk population to make informed decisions that do not further compromise their health.
